# Microbial epidemiology and antimicrobial resistance patterns of wound infection in Ethiopia: a meta-analysis of laboratory-based cross-sectional studies

**DOI:** 10.1186/s40360-019-0315-9

**Published:** 2019-05-30

**Authors:** Mekonnen Sisay, Teshager Worku, Dumessa Edessa

**Affiliations:** 1 Department of Pharmacology and Toxicology, School of Pharmacy, College of Health and Medical Sciences, Haramaya University, P.O.Box, 235, Harar, Ethiopia; 2 Department of Nursing, School of Nursing and Midwifery, College of Health and Medical Sciences, Haramaya University, P.O. Box, 235, Harar, Ethiopia; 3 Department of Clinical Pharmacy, School of Pharmacy, College of Health and Medical Sciences, Haramaya University, P.O. Box, 235, Harar, Ethiopia

**Keywords:** Wound infections, Bacteria, Antimicrobial resistance, Ethiopia

## Abstract

**Background:**

Wound infections are responsible for significant human morbidity and mortality worldwide. Specifically, surgical site infections are the third most commonly reported nosocomial infections accounting approximately a quarter of such infections. This systematic review and meta-analysis is, therefore, aimed to determine microbial profiles cultured from wound samples and their antimicrobial resistance patterns in Ethiopia.

**Methods:**

Literature search was carried out through visiting electronic databases and indexing services including PubMed, MEDLINE, EMBASE, CINAHL, and Google Scholar. Original records, available online from 2000 to 2018, addressing the research question and written in English were identified and screened. The relevant data were extracted from included studies using a format prepared in Microsoft Excel and exported to STATA 15.0 software for analyses of outcome measures and subgrouping. Der-Simonian-Laird’s random effects model was applied for pooled estimation of outcome measures at 95% confidence level. Comprehensive meta-analysis version-3 software was used for assessing publication bias across studies. The study protocol is registered on PROSPERO with reference number ID: CRD42019117638.

**Results:**

A total of 21 studies with 4284 wound samples, 3012 positive wound cultures and 3598 bacterial isolates were included for systematic review and meta-analysis. The pooled culture positivity was found to be 70.0% (95% CI: 61, 79%). Regarding the bacterial isolates recovered, the pooled prevalence of *S. aureus* was 36% (95% CI: 29, 42%), from which 49% were methicillin resistant strains. The pooled estimate of *E. coli* isolates was about 13% (95% CI: 10, 16%) followed by *P. aeruginosa*, 9% (95% CI: 6, 12%), *K. pneumoniae*, 9% (95% CI: 6, 11%) and *P. mirabilis*, 8% (95% CI: 5, 11%). Compared to other antimicrobials, *S. aureus* has showed lower estimates of resistance against ciprofloxacin, 12% (95% CI: 8, 16%) and gentamicin, 13% (95% CI: 8, 18%). *E. coli* isolates exhibited the highest point estimate of resistance towards ampicillin (*P* = 84%; 95% CI: 76, 91%). Gentamicin and ciprofloxacin showed relatively lower estimates of resistance with pooled prevalence being 24% (95% CI: 16, 33%) and 27% (95% CI: 16, 37%), respectively. Likewise, *P. aeruginosa* showed the lowest pooled estimates of resistance against ciprofloxacin (*P* = 16%; 95% CI: 9, 24%).

**Conclusion:**

Generally, the wound culture positivity was found very high indicating the likelihood of poly-microbial contamination. *S. aureus* is by far the most common bacterial isolate recovered from wound infection. The high estimate of resistance was observed among β-lactam antibiotics in all bacterial isolates. Ciprofloxacin and gentamicin were relatively effective in treating wound infections with poly-microbial etiology.

**Electronic supplementary material:**

The online version of this article (10.1186/s40360-019-0315-9) contains supplementary material, which is available to authorized users.

## Background

Wound provides a warm, moist, and nutritive environment which is conducive to microbial colonization, proliferation, and infection. It can occur during trauma, accident, burn, surgical procedures or as a result of chronic disease conditions such as diabetes mellitus and leprosy [1, 2]. All wounds can be contaminated from endogenous sources of the patient (e.g. nasopharynx and gastrointestinal tract), the surrounding skin and/or the immediate environment. The local environment is particularly important for patients admitted in healthcare settings. Microbes in such wounds create a continuum from initial contamination all the way through colonization to fully blown infection. For this infection to occur, factors including the virulence characteristics of microbes, selection pressures, the host immune system, age and comorbid conditions of a patient play a critical role. Skin serves as a first line defense (innate immunity) in battle against microbes. Despite this, wound breaks the integrity of the skin, and creates an open filed for microbes thereby circumvents the innate immunity and establishes infection. It is crystal clear that wound infections have resulted in considerable morbidity, mortality, prolonged hospitalization and escalation of direct and indirect healthcare costs [3, 4].

Though it varies based on the wound source, the most commonly isolated gram-positive cocci are *Staphylococcus aureus* and *Coagulase Negative Staphylococci* (CoNS). Besides, gram-negative aerobic bacilli such as *Escherichia coli*, *Pseudomonas aeruginosa*, *Klebsiella pneumoniae* and *Proteus mirabilis* are the most prevailing clinically relevant isolates [5–9]. Among these isolates, *S. aureus* particularity Methicillin resistant strains (MRSA) as well as *P. aeruginosa* are typical biofilm producers making the wound infection difficult to treat using standard antibiotics [3, 10–12].

Treatment of wound infections with antimicrobial agents as well as optimum treatment regimens remains ill defined. Many published guidelines are mainly based on expert opinion rather than evidence-based data. The selection of appropriate antimicrobial agents has been inconclusive. Though prophylactic use of antimicrobials can help reduce the risk of infection and promotes wound healing, it is not a direct substitute for good local wound care such as irrigation and surgical debridement. Moreover, judicious use of antimicrobials reduces the development of antimicrobial resistance (AMR) [13–15]. Review of recent practices has revealed that potentially inappropriate and inconsistent use of antimicrobials following surgical procedures contributes to development of AMR. In addition, appropriateness of the timing, the duration, route and selection of these agents remains elusive [13, 16–18]. this systematic review and meta-analysis is aimed to provide nationwide pooled estimates of wound culture positivity, microbial profiles and AMR patterns of wound infection in Ethiopia. This will help as a benchmark for developing antimicrobial stewardship programs and generating evidence-based selection of antimicrobials thereby preserve the available antimicrobials and contain AMR.

## Methods

### Study protocol

The identification and screening of studies as well as eligibility assessment of full texts was conducted in accordance with the preferred reporting items for systematic review and meta-analysis (PRISMA) statement [19]. In addition, the content of this meta-analysis is well described in the completed PRISMA check list [20] (Additional file 1: Table S1). The study protocol is registered on International Prospective Register of Systematic Reviews (PROSPERO) with reference number ID: CRD42019117638 and available online at: http://www.crd.york.ac.uk/PROSPERO/display_record.php?ID=CRD42019117638

### Data sources and searching strategy

Literature search was carried out through visiting electronic databases and indexing services. The PubMed, MEDLINE (Ovid®), EMBASE (Ovid®), CINAHL (EBSCOhost), and Google Scholar, were used as main source of data. Besides, other supplementary sources including ResearchGate, WorldCat, Science Direct and University repositories were searched to retrieve relevant data. Excluding the non-explanatory terms, the search strategy included important keywords and indexing terms: wound, wound infection (MeSH), “antimicrobial susceptibility”, bacteria (MeSH), “antimicrobial resistance”, “antibacterial resistance”, and “Ethiopia”. The MeSH terms, Boolean operators (AND, OR and NOT), and truncation were applied for appropriate searching and identification of records for the research question.

### Inclusion and exclusion criteria

#### Inclusion criteria

- Studies conducted in Ethiopia
- Studies available online from 2000 to 2018
- Studies published in English language
- Laboratory-based observational (e.g. cross-sectional) studies addressing the research question: studies dealing with wound culture positivity and prevalence of individual isolates recovered from wound samples as primary outcome measures

#### Exclusion criteria

- Any review papers published in the study period
- Studies having mixed sample sources (wound and non-wound samples concurrently. E.g. blood, urine, and other discharges)
- Wound samples taken from animals
- Irretrievable full texts (after requesting full texts from the corresponding authors via email and/or Research Gate account)
- Studies with unrelated or insufficient outcome measures
- Studies with outcomes of interest are missing or vague

### Screening and eligibility of studies

Along with application of appropriate limits, online records from each database or directory were exported to ENDNOTE reference software version 8.2 (Thomson Reuters, Stamford, CT, USA). The records were then merged to one folder to identify and remove duplicates with the help of ENDNOTE and/or manual tracing as there is a possibility of several citation styles per study. Thereafter, two authors Mekonnen Sisay (MS) and Dumessa Edessa (DE) independently screened the title and abstracts with the predefined inclusion/exclusion criteria. Records which passed the screening phase were subjected for eligibility assessment of full texts. For this, two authors, MS and Teshager Worku (TW) independently collected full texts and evaluated their eligibility for meta-analysis. In each case, the third author was consulted to solve disagreement occurred between the two authors.

### Data extraction

Using standardized data abstraction format prepared in Microsoft Excel (Additional file 2: Table S2), the authors independently extracted important data related to study characteristics (study area, first author, year of publication, study design, population characteristics, nature of wound samples) and outcome of interests (culture positivity, nature of bacterial isolates, prevalence of bacterial isolates and resistance profile of individual isolates).

### Critical appraisal of studies

The quality of studies was assessed using standard critical appraisal tools prepared by Joanna Briggs Institute (JBI), at University of Adelaide, Australia [21]. The purpose of this appraisal is to assess the methodological quality of a study and to determine the extent to which a study has addressed the possibility of bias in its design, conduct and analysis. The JBI critical appraisal checklist for studies reporting prevalence data contains nine important questions (Q1-Q9) and primarily addresses the methodological aspects of each study. This critical appraisal was conducted to assess the internal (systematic error) and external validity of studies thereby reduces the risk of biases among individual studies. Scores of the two authors (MS and DE) in consultation with third author (TW) (in case of disagreement between the two authors’ appraisal result) were taken for final decision. Studies with the number of positive responses (yes) greater than half of the number of checklists (i.e., score of five and above) were included in the systematic review and meta-analysis.

### Outcome measurements

The primary outcome measures are the culture positivity of wound samples and the prevalence of bacterial isolates recovered from infected wound samples in Ethiopia. The secondary outcome measure is the antimicrobial resistance profiles of clinically relevant bacterial isolates against commonly prescribed antimicrobial agents taken from different pharmacologic categories (penicillins, cephalosporins, aminoglycosides, fluroquinolones, macrolides and sulfonamides). Subgroup analysis was also conducted based on the nature of wound sources (surgical, burn and other non-surgical wounds).

### Data processing and analysis

The extracted data were imported from Microsoft Excel to STATA software, version 15.0 for the pooled estimation of outcome measures. Sensitivity and subgroup analyses were also conducted to minimize the degree of heterogeneity. Der-Simonian-Laird’s random effects model was applied for the analysis at 95% confidence level. Heterogeneity among the included studies was assessed with I^2^ statistics. Forest plots and summary tables were used to report the results. Comprehensive Meta-analysis software version-3, (Biostat, Englewood, New Jersey, USA), was employed for assessment of publication bias. The presence of publication bias was evaluated by using Egger’s regression and Begg’s and Mazumdar’s correlation tests and presented with funnel plot. All statistical tests with *p*-values less than 0.05 (one-tailed) were considered significant [22, 23].

## Results

### Search results

**Fig. 1:**
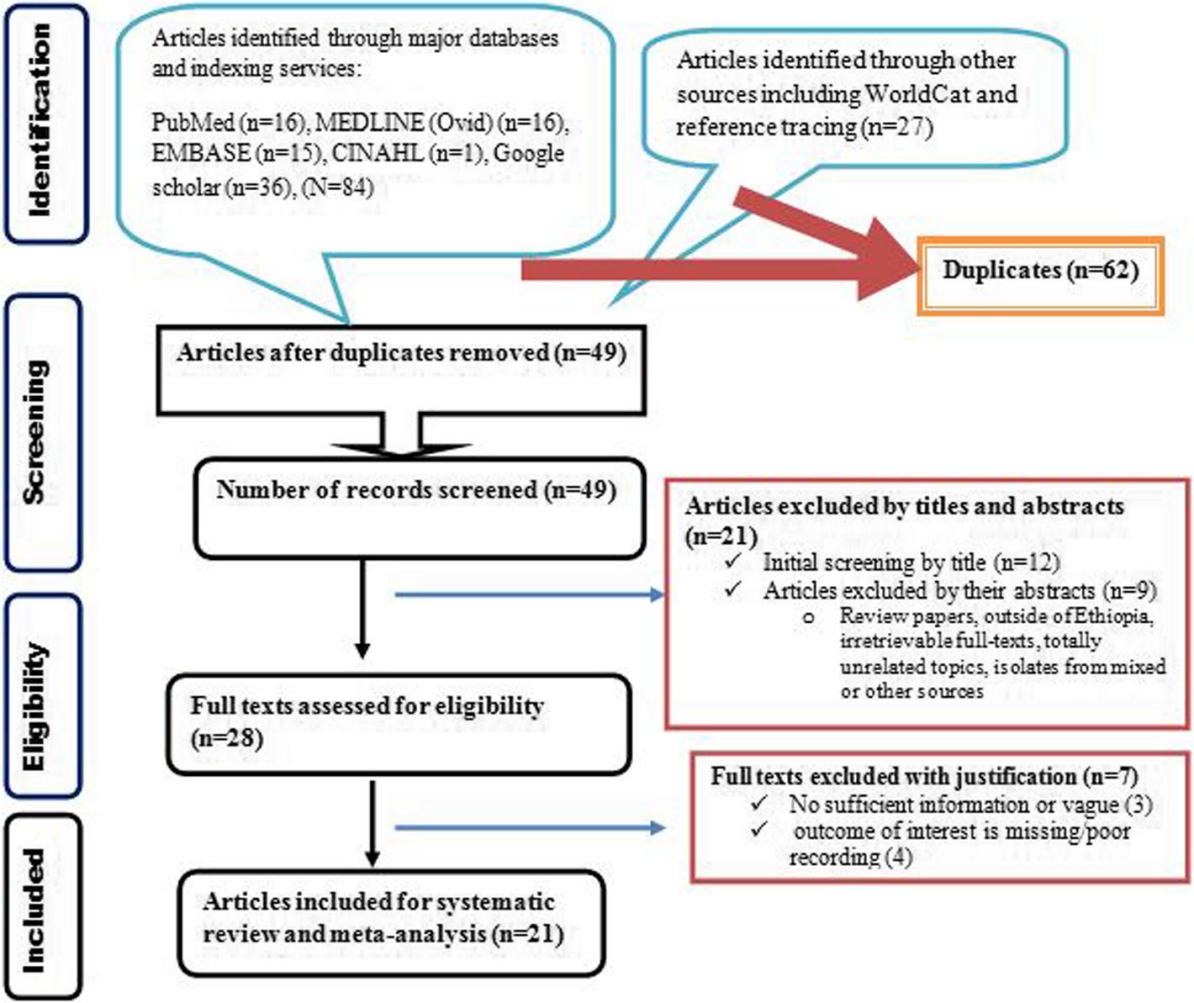
PRISMA flow diagram depicting the selection process

A literature search in electronic databases including PubMed, MEDLINE, EMBASE, CINAHL and Google Scholar retrieved a total of 111 studies. From which, 62 studies were found duplicate through ENDNOTE and manual tracing. The remaining studies were screened using their titles and abstracts and 21 of them did not fulfill the inclusion criteria and thus removed from further eligibility study. The full texts of 28 studies were thoroughly assessed to ensure the presence of at least the primary outcome measures in sufficient and non-ambiguous way. In this regard, 7 studies did not meet the inclusion criteria and thus removed from final inclusion. Therefore, 21 studies addressing the outcome of interest were included (Fig. 1).

### Results of quality assessment

Having fulfilled the predefined inclusion criteria, further rigorous appraisal of individual study was conducted using JBI checklist with the average quality score ranging between 6 and 9. Finally, the 21 studies were included for systematic review and meta-analysis (Table 1).

**Table 1:** Quality assessment of studies using JBI’s critical appraisal tools designed for cross-sectional studies

Studies	JBI’s critical appraisal questions									Overall score	Include
	Q1	Q2	Q3	Q4	Q5	Q6	Q7	Q8	Q9		
Abraham and Wamisho., 2009	Y	Y	Y	Y	Y	Y	Y	U	N	7	✓
Alebachew et al., 2012	N	Y	N	Y	N	Y	Y	Y	Y	6	✓
Asres et al., 2017	N	Y	Y	Y	Y	Y	Y	Y	Y	8	✓
Azene et al., 2011	U	Y	Y	Y	Y	Y	Y	Y	Y	8	✓
Bitew et al., 2018	U	Y	Y	Y	Y	Y	Y	Y	Y	8	✓
Desalegn et al., 2014	Y	Y	Y	Y	Y	Y	Y	N	Y	8	✓
Dessie et al., 2016	U	Y	N	Y	Y	Y	Y	Y	Y	7	✓
Gelaw et al., 2014	U	Y	N	Y	Y	Y	Y	Y	Y	7	✓
Godebo et al., 2013	U	Y	Y	Y	Y	Y	Y	Y	Y	8	✓
Guta et al., 2014	N	Y	N	Y	Y	Y	Y	Y	Y	7	✓
Hailu et al., 2016	U	Y	Y	Y	Y	Y	Y	N	Y	7	✓
Kahsay et al., 2014	Y	Y	Y	Y	Y	Y	Y	Y	Y	9	✓
Kiflie et al., 2018	Y	Y	N	Y	Y	Y	Y	Y	Y	8	✓
Lema et al., 2012	N	Y	Y	Y	Y	Y	Y	Y	Y	8	✓
Mama et al., 2014	Y	Y	Y	Y	Y	Y	Y	Y	Y	9	✓
Mengesha et al., 2014	Y	Y	N	Y	Y	Y	Y	Y	Y	8	✓
Mohammed et al., 2014	N	Y	N	Y	Y	Y	Y	Y	Y	7	✓
Mulu et al., 2006	U	Y	Y	Y	Y	Y	Y	Y	Y	8	✓
Mulu et al., 2017	U	Y	Y	Y	Y	Y	Y	Y	Y	8	✓
Sewnet et al., 2013	N	Y	N	Y	Y	Y	Y	Y	Y	7	✓
Tekie, 2008	Y	Y	Y	Y	Y	Y	Y	Y	Y	9	✓

*Y* Yes, *N* No, *U* Unclear, *Q* Question. Overall score is calculated by counting the number of Ys in each row

Q1 = Was the sample frame appropriate to address the target population? Q2 = Were study participants sampled in an appropriate way? Q3 = Was the sample size adequate? Q4 = Were the study subjects and the setting described in detail? Q5 = Was the data analysis conducted with sufficient coverage of the identified sample? Q6 = Were valid methods used for the identification of the condition? Q7 = Was the condition measured in a standard, reliable way for all participants? Q8 = Was there appropriate statistical analysis? Q9 = Was the response rate adequate, and if not, was the low response rate managed appropriately?

### Study characteristics

Twenty one studies with a total of 4284 wound samples, 3012 positive cultures and 3598 bacterial isolates were included for systematic review and meta-analysis. This review included a wide range of wound samples taken from various patient sources including patients surgical wounds [24–32], patients with non-surgical and/or combined wound infections [33–40], patients with fracture [41], patients with burn [42, 43], and patients with leprotic wound infections [44]. The study period of included studies ranges from 2000 to 2018 and three of which were published before 2010 [30, 39, 41]. Regarding the study design, all of them are laboratory based cross-sectional studies with four of them being a retrospective chart review [33, 36, 39, 40]. The number of wound samples, positive cultures and bacterial isolates ranges from 50 [43] to 599 [33], 21 [43] to 422 [33] and 47 [43] to 500 [33], respectively. Two studies have only recovered *S. aureus* isolates from positive cultures of wound swabs [28, 42]. With exception of three studies which were conducted in regional laboratories [33, 34, 36], all the rest studies were conducted in specialized and/or teaching hospitals of Ethiopian universities. Table 2 has also summarized the number of clinically relevant gram-positive (*S. aureus* and CoNS) and gram-negative (*E. coli*, *P. aeruginosa*, *K. pneumoniae*, and *P. mirabilis*) bacterial isolates recovered from positive cultures of wound samples (Table 2).

**Table 2:** Characteristics of included studies describing the magnitude of culture positive wound samples and microbial profiles of clinical relevant bacterial isolates in Ethiopia (2000–2018)

Studies	Year of publication	Study setting	Total patients (M/F ratio)	Study characteristics	Wound samples	Culture positive	No of isolates	Gram-positive		Gram -negative			
								*S. aureus*	*CoNS*	*E. coli*	*P. aeruginosa*	*K. pneumoniae*	*P. mirabilis*
Abraham and Wamisho [41]	2009	TASH, AA	191 (158/33)	Fracture in-and outpatients	200	196	162	24	12	17	16	12	6
Alebachew et al. [42]	2012	Yekatit 12 hospital	114 (58/56)	Burn in-and outpatients	114	95	114	65	ND	ND	ND	ND	ND
Asres et al. [24]	2017	TASH, AA	197 (118/79)	Postoperative in-and outpatients	197	149	168	56	19	24	8	15	2
Azene et al. [33]	2011	Dessie regional laboratory	12599 (368/231)	Outpatients with any wound	599	422	500	208	9	82	92	12	55
Bitew et al. [34]	2018	Arsho medical laboratory	366 (213/153)	Both in-and Patients with wound	366	271	271	110	11	49	14	12	7
Desalegn et al. [25]	2014	Hawassa TRH	194 (116/78)	Post-surgical in and outpatients	194	138	177	66	6	45	18	24	18
Dessie et al. [31]	2016	St. Paul and Yekatit 12 hosp.	107 (56/51)	Surgical inpatients	107	90	104	19	4	24	6	10	1
Gelaw et al. [26]	2014	UoG TH	42 (27/15)	Surgical inpatients	142	42	49	11	4	6	3	10	9
Godebo et al. [35]	2013	JUSH	NS	Out/inpatients with wound	322	310	384	73	14	30	74	46	107
Guta et al. [27]	2014	HUTRH	100 (37/63)	Surgical inpatients	100	92	177	45	26	30	16	32	12
Hailu et al. [36]	2016	Bahir Dar RHRL	234 (131/103)	Both in-and outpatients with wound	380	234	234	100	ND	33	26	20	22
Kahsay et al. [28]	2014	DMRH	184 (61/123)	Surgical inpatients	184	73	184	72	ND	ND	ND	ND	ND
Kiflie et al. [32]	2018	UoG TH	107 (0/107	Women with cesarean section or episiotomy	107	90	101	42	13	20	1	14	1
Lema et al. [44]	2012	Selected Hospitals, AA	245 (157/88)	In-and outpatients with leprosy	245	222	298	68	18	14	7	2	47
Mama et al. [37]	2014	JUSH	150 (107/43)	In/outpatients with wound	150	131	145	47	21	29	11	14	23
Mengesha et al. [29]	2014	Ayder TRH	128 (98/30)	Surgical inpatients	128	96	123	40	18	6	11	29	15
Mohammed et al. [38]	2014	UoG RH	137 (81/56)	In/outpatients with wound	137	115	136	39	17	8	8	17	6
Mulu et al. [39]	2006	UoGTH	NS	In/outpatients with wound	151	79	79	51	ND	8	ND	7	3
Mulu et al. [40]	2017	DMRH	238 (NS)	In/outpatients with wound	238	115	90	70	ND	5	6	1	3
Sewnet et al. [43]	2013	Yekatit 12 hospital	50 (30/20)	In/outpatients with burn case	50	21	47	16	6	ND	15	1	4
Tekie [30]	2008	TASH	173 (97/76)	Outpatients with surgical wound	173	31	55	14	11	7	8	4	2
Total					4284	3012	3598						

*M* Male, *F* Female, *CoNS* Coagulase negative Staphylococci, *ND* Not determined, *JUSH* Jimma University Specialized Hospital, *UoGTH* University of Gondar Teaching Hospital, Hawassa University Teaching and Referral Hospital, *CS* Cross-sectional, *R* Retrospective, *TASH* Tikur Anbesa Specialized Hospital, *AA* Addis Ababa, *DMRH* Debre Markos Referral Hospital, *NS* Not specified

Besides, the antimicrobial resistance profiles of six clinical isolates (two from gram-positive and four from gram-negative bacteria) were summarized against commonly prescribed antimicrobial agents taken from various pharmacologic classes: penicillins (amoxicillin, ampicillin, amoxicillin-clavulanic acid, and methicillin), cephalosporins (ceftriaxone), fluoroquinolones (ciprofloxacin), marcrolides (erythromycin), sulfonamides (cotrimoxazole) and aminoglycosides (gentamicin). Unlike others, a study conducted by Hailu et al. utilized variable number of bacterial isolates per antimicrobial agent [36]. Antimicrobial susceptibility testing of gram-negative isolates was conducted against erythromycin in two studies [29, 33] though not found suitable for meta-analysis (Table 3).

**Table 3:** Antimicrobial resistance patterns of clinically relevant bacterial isolates from wound infection in Ethiopia

Types of Bacteria	Studies	Number of isolates	Number of isolates resistant to								
			AMO	AMC	AMP	CIP	CRO	SXT	ERY	GEN	MET
*S. aureus*	Abraham and Wamisho	24	9	6	20	4	2	6	2	3	5
	Alebachew et al	66	–	22	–	–	23	–	9	–	51
	Asres et al.	56	–	9	–	7	–	19	9	7	6
	Azene et al	208	165	–	–	18	37	140	72	26	22
	Bitew et al	110	–	–	–	7	–	30	70	2	–
	Kiflie et al	42	28	–	30	–	15	26	–	–	22
	Desalegn et al	66		20	63	26	54	37	29	26	–
	Dessie et al	19	–	–	–	3	–	4	4	3	–
	Godebo et al	73	–	–	67	10	15	44	58	12	57
	Guta et al	45	45	–	–	–	16	–	–	9	–
	Hailu et al	NDA	–	–	–	5(67)	–	16 (94)	30 (96)	–	20 (95)
	Kahsay et al	73	13	–	13	–	–	1	–	9	36
	Lema et al	68	45	8	58	6	–	23	20	5	50
	Mama et al	47	–	–	45	2	7	3	7	2	–
	Mengesha et al	40	37	20	36	–	36	–	9	4	34
	Mohammed et al	39	34	–	–	8	8	15	24	7	30
	Mulu et al., 2006	51	–	–	28	–	–	18	–	–	–
*CoNS*	Abraham and Wamisho	12	1	–	4	1	1	–	1	2	
	Asres et al.	19	–	15	–	5	–	14	11	12	
	Kiflie et al	13	11	–	11	–	5	8	–	–	
	Desalegn et al	6	–	3	6	3	6	3	3	3	
	Dessie et al	4	–	–	–	4	–	3	3	3	
	Godebo et al	14	–	–	9	0	2	5	0	0	
	Guta et al	26	4	–	–	–	13	–	–	13	
	Lema et al	18	6	–	12	1	–	9	9	3	
	Mama et al	21	–	–	19	5	6	3	8	4	
	Mengesha et al	18	16	14	14	–	13	–	9	9	
	Mohammed et al	16	13	–	–	3	8	7	8	3	
*E. coli*	Abraham and Wamisho	17	8	4	7	1	2	5	–	2	
	Asres et al.	24	20	14	–	5	–	15	–	7	
	Azene et al	82	70	–	–	21	55	55	43	12	
	Bitew et al	49	–	35	35	5	10	22	–	5	
	Kiflie et al	20	14	–	16	4	12	10	–	7	
	Desalegn et al	45	–	21	45	18	18	27	–	21	
	Dessie et al	24	–	17	23	16	20	–	–	13	
	Godebo et al	30	–	–	23	1	20	6	–	0	
	Guta et al	30	20	–	–	–	10	–	–	0	
	Hailu et al	NDA	–	24 (33)	31 (33)	15 (33)	6 (24)	23 (30)	–	18 (33)	
	Lema et al	14	10	7	9	–	–	9	–	–	
	Mama et al	29	–	–	29	10	18	16	–	15	
	Mengesha et al	6	4	6	6	1	4	–	2	–	
	Mohammed et al	8	–	–	6	3	1	4	–	1	
	Mulu et al., 2006	8	–	–	7	–	–	5	–	–	
*P. aeruginosa*	Abraham and Wamisho	16	13	13	14	0	5	11	–	2	
	Asres et al.	8	8	7	–	1	–	7	–	2	
	Azene et al	92	76	–	–	4	47	71	83	7	
	Bitew et al	14	–	14	14	1	11	9	–	1	
	Desalegn et al	18	–	18	18	0	18	12	–	9	
	Dessie et al	6	–	6	6	2	5	–	–	0	
	Godebo et al	74	–	–	72	4	7	65	–	8	
	Guta et al	16	16	–	–	–	8	–	–	8	
	Hailu et al	NDA	–	–	–	5 (26)	–	3 (9)	–	7 (23)	
	Lema et al	7	6	7	6	–	–	7	–	1	
	Mama et al	11	–	–	11	–	7	8	–	2	
	Mengesha et al	11	11	11	11	9	11	–	3	–	
	Mohammed et al	8	–	–	–	3	3	–	–	3	
	Tekie	8	–	–	–	6	4	7	–	3	
*K. pneumoniae*	Abraham and Wamisho	12	8	4	12	0	3	3	–	3	
	Asres et al.	15	14	12	–	4	13	13	–	9	
	Azene et al	12	12	–	–	–	–	8	–	0	
	Bitew et al	12	–	12	12	2	7	5	–	2	
	Kiflie et al	14	14	–	14	2	8	9	–	3	
	Desalegn et al	24	–	12	21	15	21	21	–	24	
	Dessie et al	10	–	10	10	2	9	–	–	4	
	Godebo et al	46	–	–	32	13	13	30	–	13	
	Guta et al	32	25	–	–	–	9	–	–	12	
	Hailu et al	NDA	–	10 (20)	15 (20)	4 (20)	2 (18)	8 (20)	–	11 (18)	
	Mama et al	14	–	–	14	5	10	12	–	9	
	Mengesha et al	29	29	19	26	11	25	–	13	8	
	Mohammed et al	17	–	–	16	10	9	11	–	5	
*P. mirabilis*	Abraham and Wamisho	6	2	1	1	0	0	3	–	0	
	Azene et al	55	48	–	–	9	35	45	51	5	
	Bitew et al	7	–	4	5	2	5	5	–	2	
	Desalegn et al	18	–	9	18	0	6	3	–	6	
	Godebo et al	107	–	–	77	8	8	81	–	35	
	Guta et al	12	11	–	–	–	8	–	–	6	
	Hailu et al	NDA	–	12 (22)	17 (22)	5 (22)	8 (18)	7 (17)	–	5 (22)	
	Lema et al	47	24	20	31	4	–	21	–	4	
	Mama et al	23	–	–	21	4	15	9	–	6	
	Mengesha et al	15	15	7	13	7	11	–	9	3	
	Mohammed et al	6	–	–	6	2	1	5	–	2	

---, Not tested, *NDA* Number of isolates is different among antimicrobial agents tested as indicated in parenthesis of the corresponding rows, *AMO* Amoxicillin, *AMP* Ampicillin, *AMC* Amoxicillin-clavulanic acid, *SXT* Cotrimoxazole, *CRO* Ceftriaxone, *CIP* Ciprofloxacin, *GEN* Gentamicin, *ERY* Erythromycin

### Outcome measures

#### Primary outcome measures: culture positivity and microbial epidemiology

**Fig. 2:**
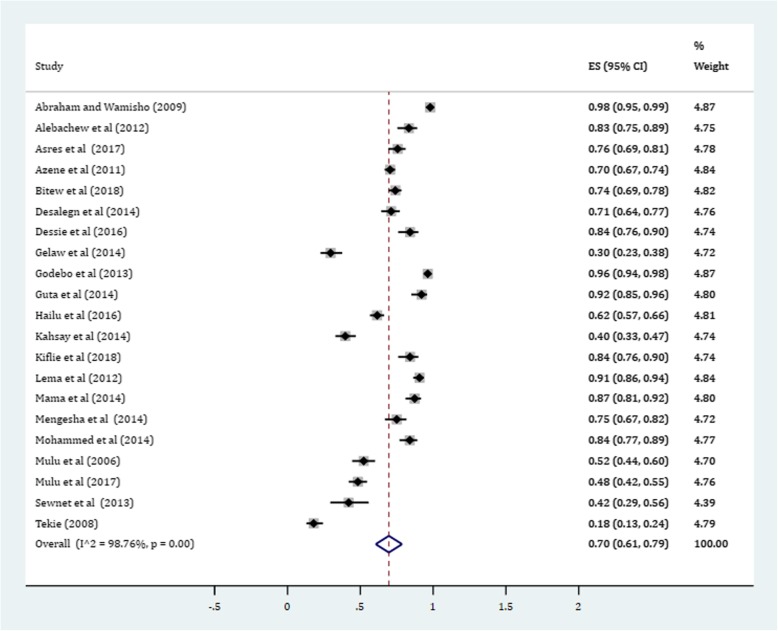
Forest plot depicting culture positivity among wound sample in Ethiopia

**Fig. 3:**
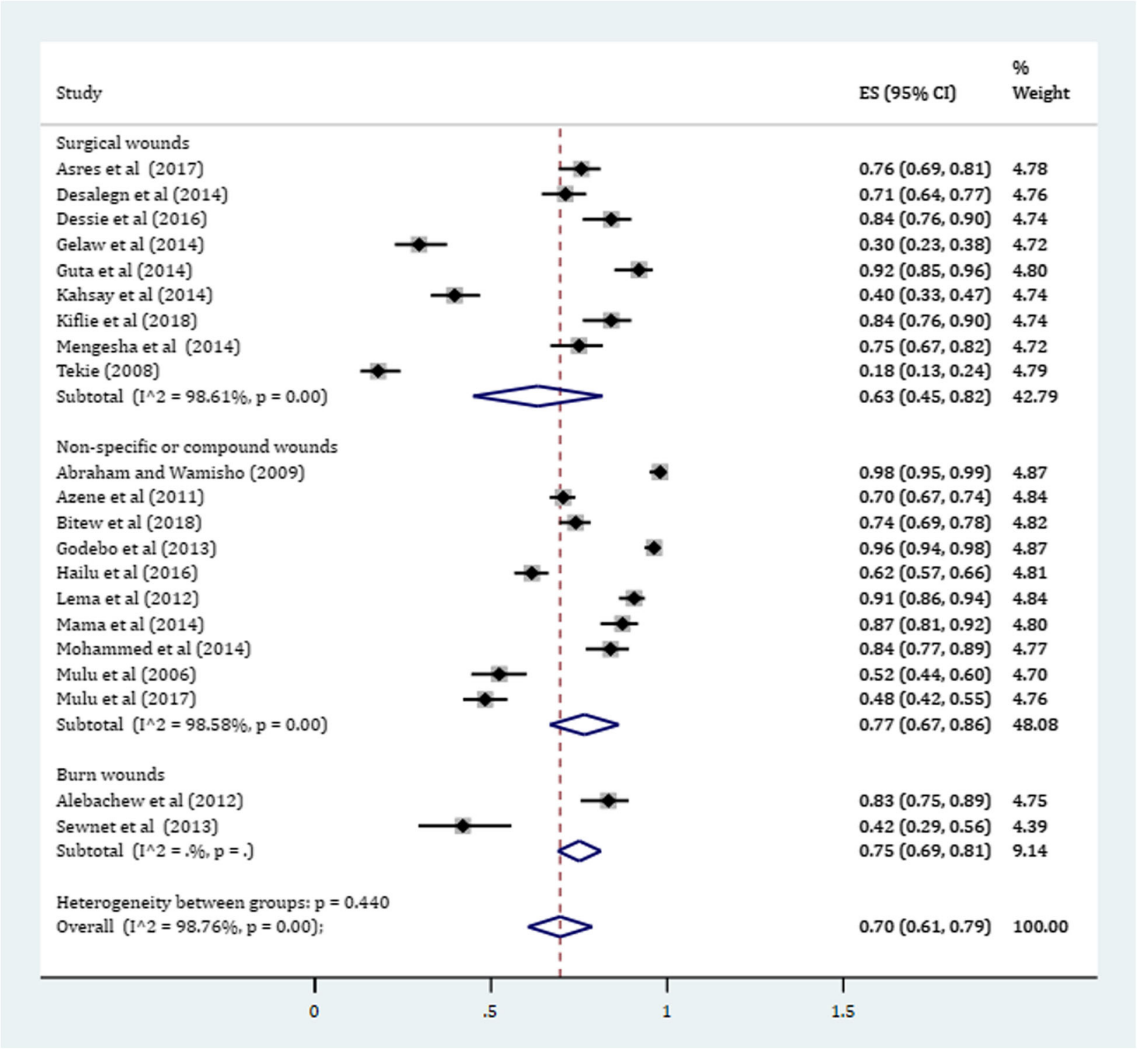
Forest plot showing subgroup analysis of culture positivity based on wound sources

In this meta-analysis, a total of 3012 positive bacterial cultures obtained from 4284 wound samples were included. The pooled prevalence of culture positive cases from all wound samples was found to be 70.0% (95% CI: 61, 79%) (Fig. 2). Subgroup analysis was conducted to determine whether there is a potential difference of culture positivity among wound sources. Based on this, the prevalence of culture positive samples in non-specific non-surgical simple or compound wounds was estimated about 77% (95% CI: 67, 86%). Likewise, the prevalence of cultures with bacterial growth among burn wounds was estimated 75% (95% CI: 69, 81%). In surgical wounds, a relatively lower culture positivity rate was obtained with a pooled estimate being 63% (95% CI: 45, 82%) (Fig. 3).

**Fig. 4:**
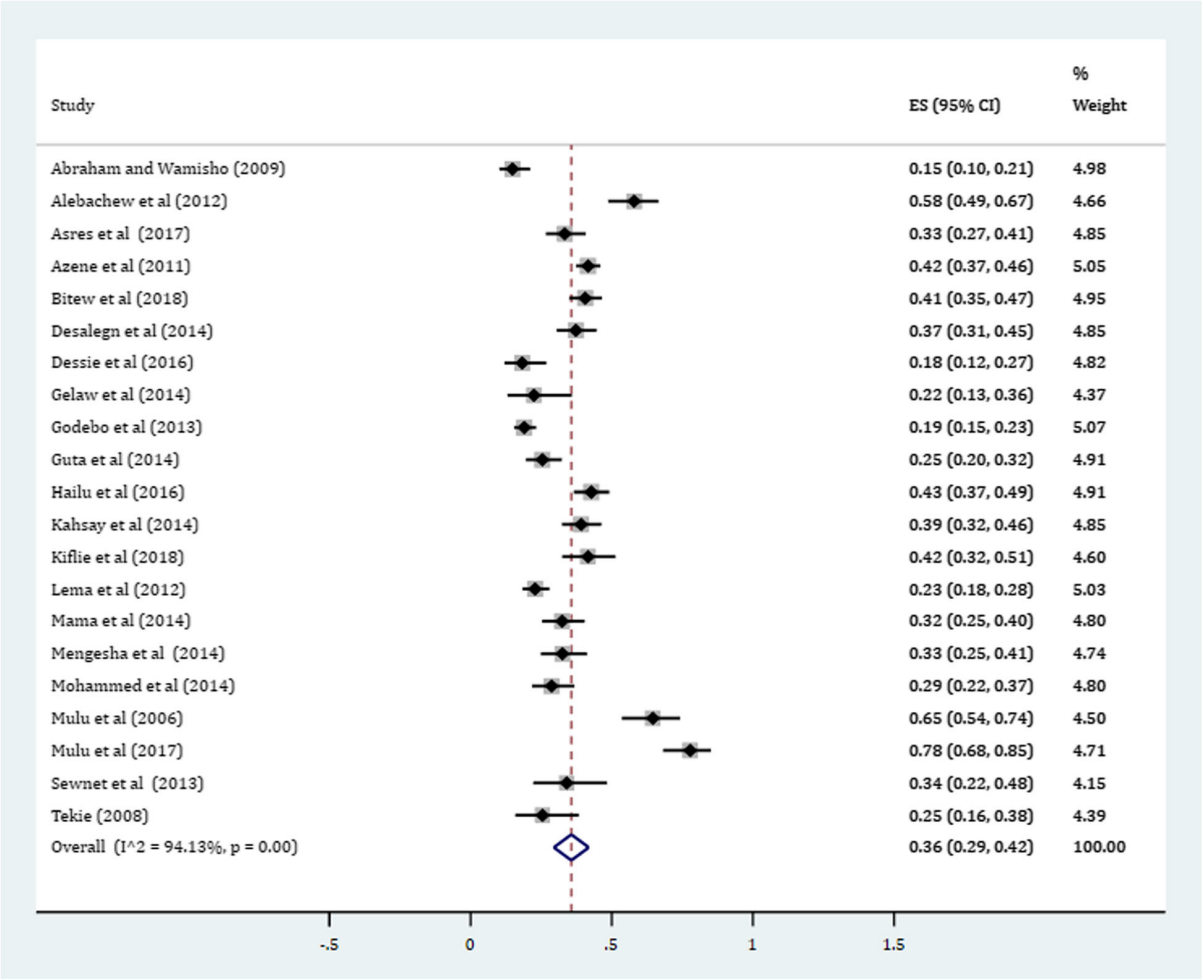
Prevalence of *S. aureus* in wound samples

**Fig. 5:**
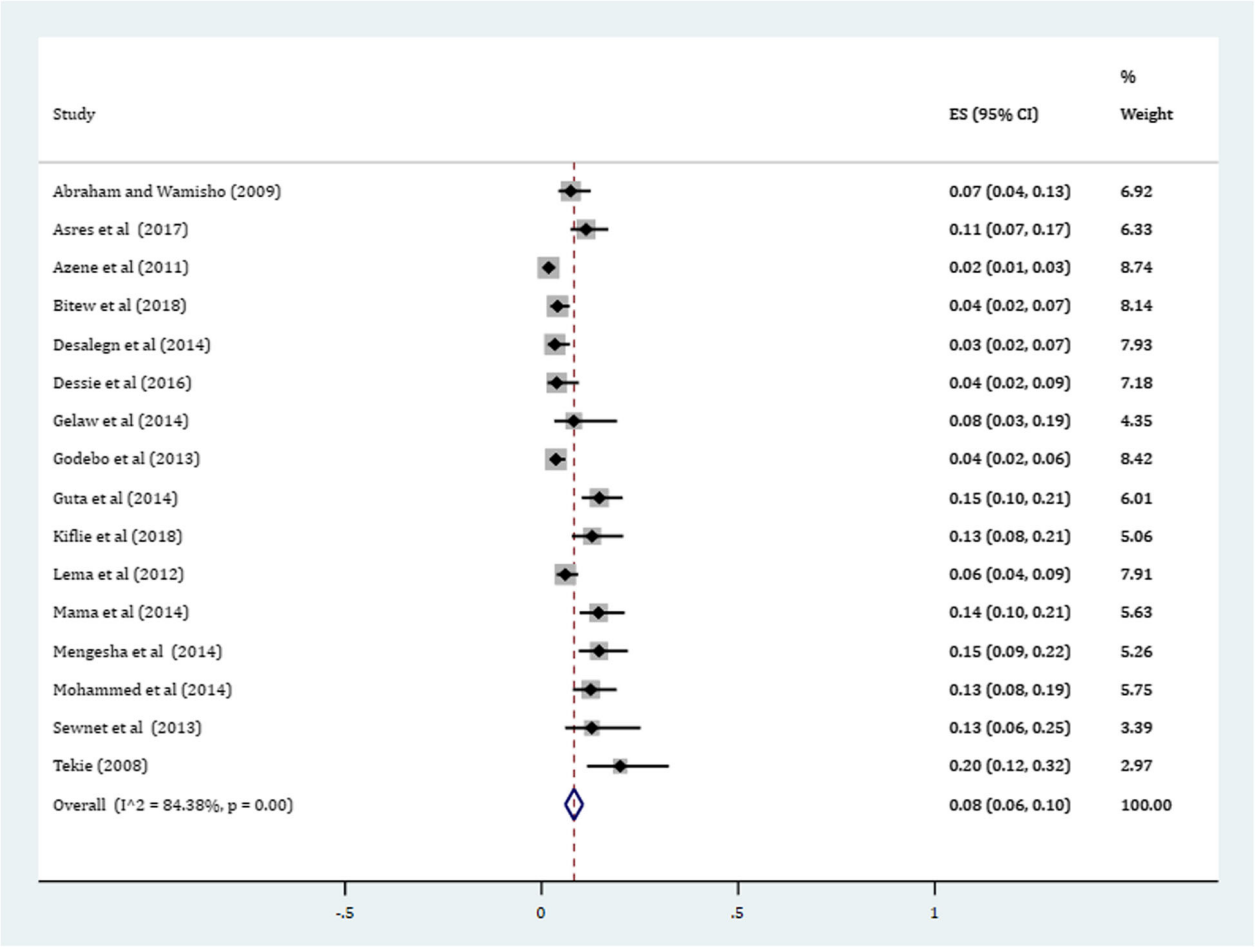
Pooled estimate of CoNS in wound samples in Ethiopia

**Fig. 6:**
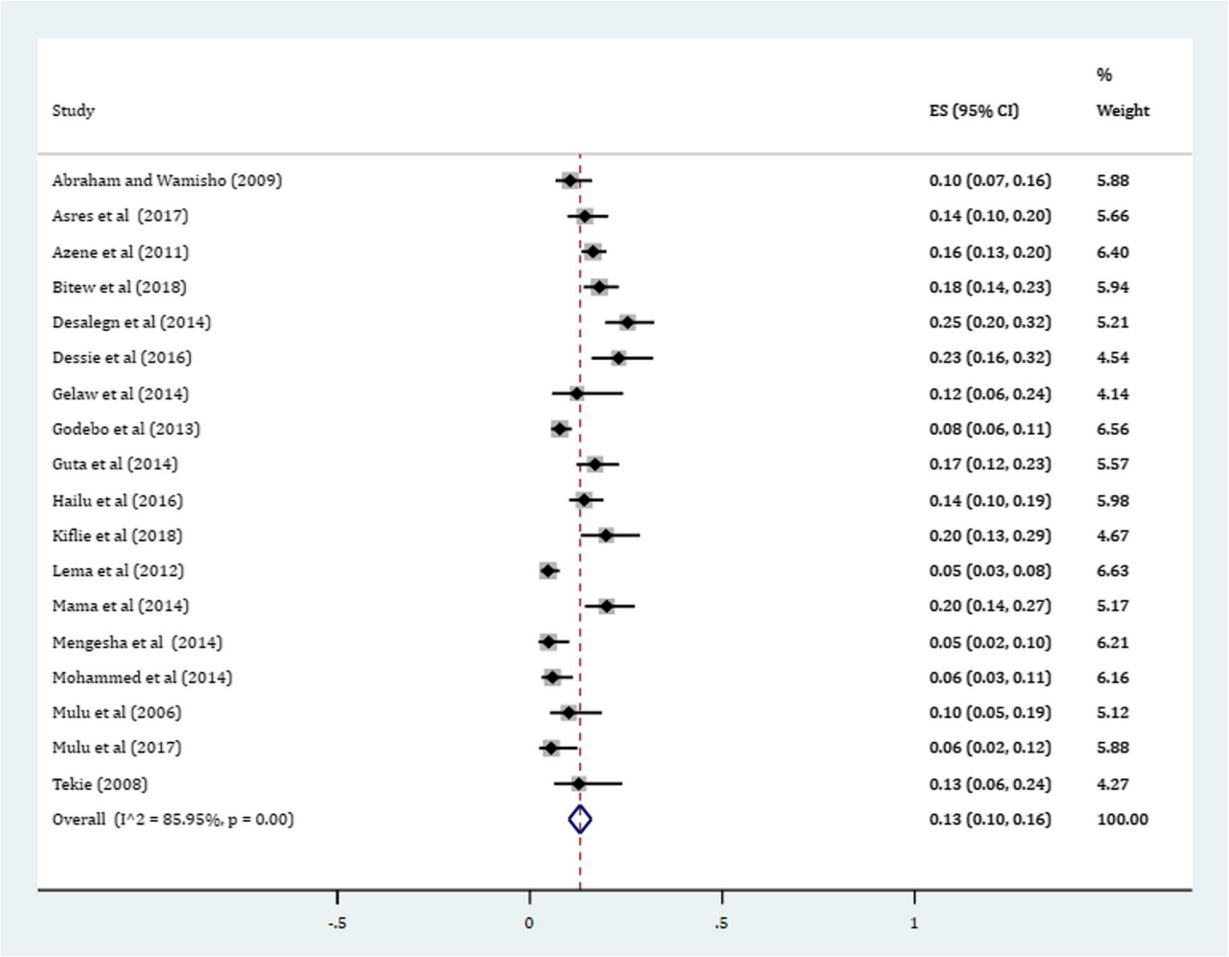
Pooled estimates of *E. coli* in wound samples

**Fig. 7:**
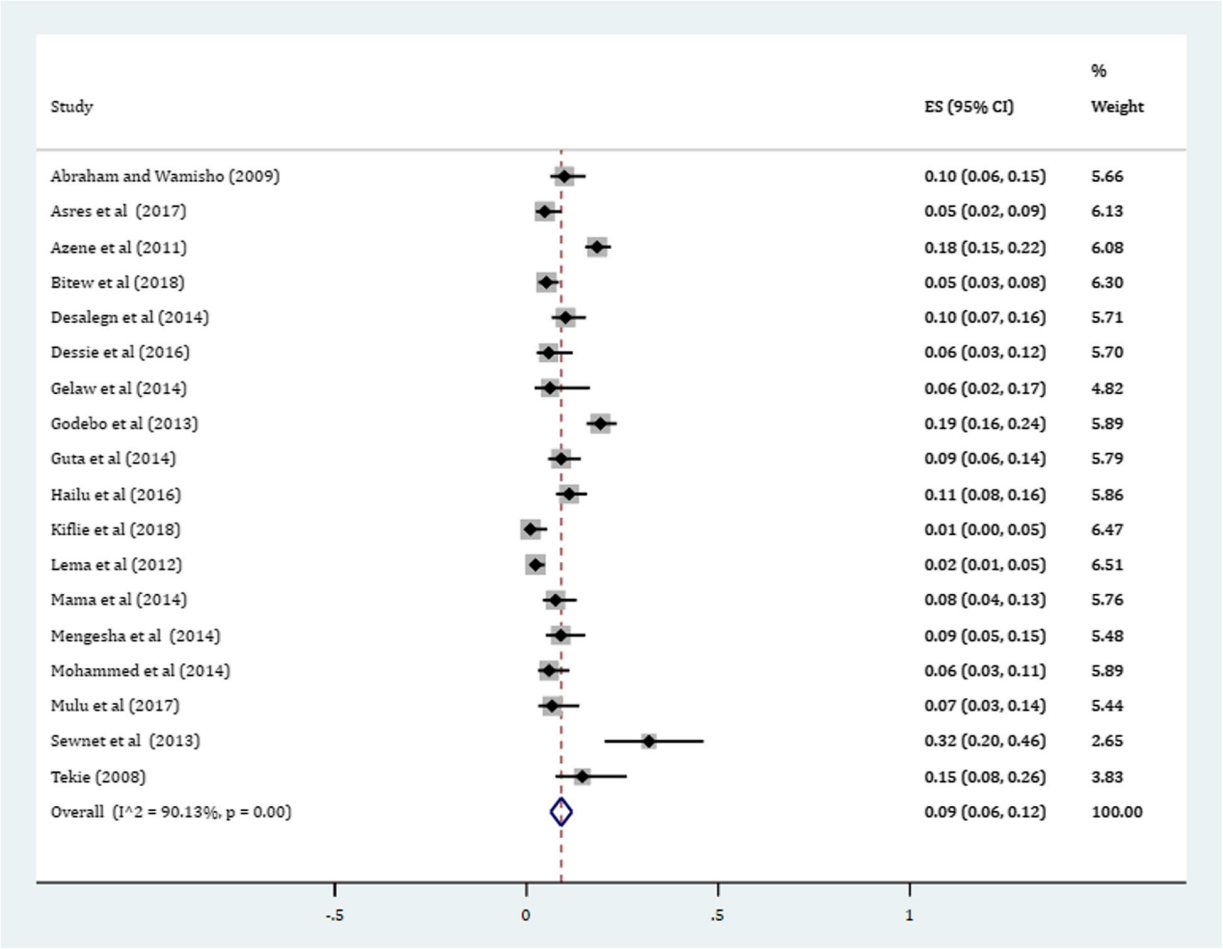
Forest plot depicting the pooled prevalence of *P. aeruginosa* in wound samples

**Fig. 8:**
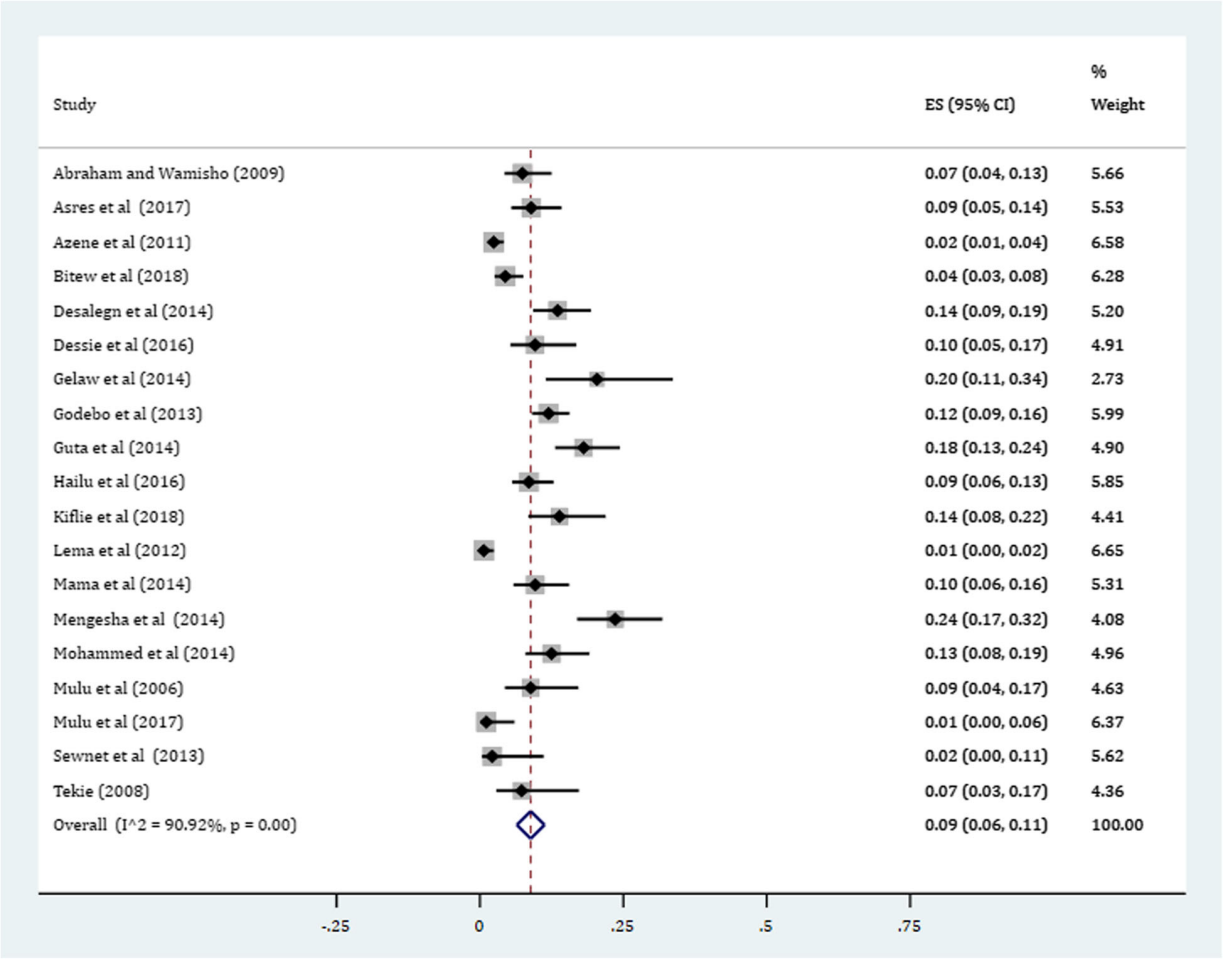
Pooled estimates of *K. pneumoniae* in wound samples

**Fig. 9:**
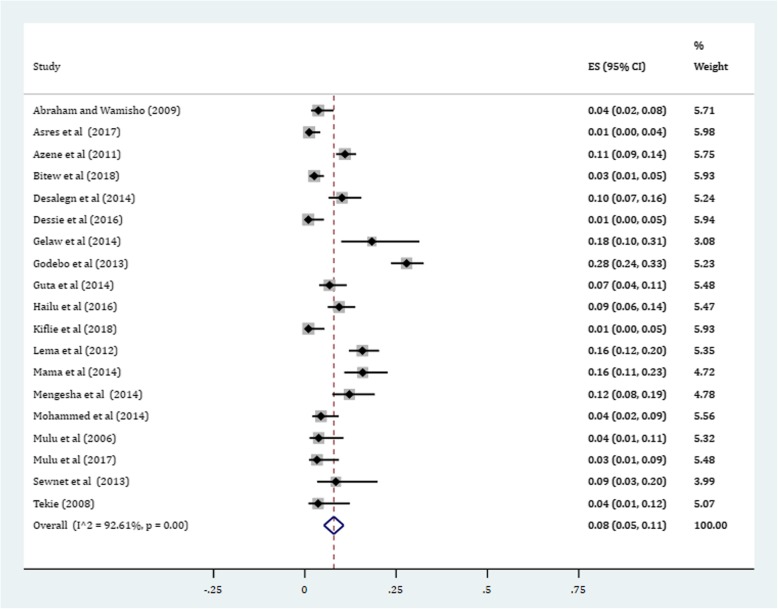
Pooled estimate of *P. mirabilis*

Gram-positive cocci were a predominant isolates recovered. The pooled prevalence of *S. aureus* was 36% (95% CI: 29, 42%) (Fig. 4). The prevalence of CoNS isolates from wound samples was estimated to be 8% (95% CI: 6, 10%) (Fig. 5). With regard to gram-negative aerobic bacilli, the pooled estimates of *E. coli* isolates from all isolates of wound samples was found to be 13% (95% CI: 10, 16%) (Fig. 6) followed by *P. aeruginosa*, 9% (95% CI: 6, 12%) (Fig. 7), *K. pneumoniae*, 9% (95% CI: 6, 11%) (Fig. 8) and *P. mirabilis*, 8% (95% CI: 5, 11%) (Fig. 9).

#### Secondary outcome measures: antimicrobial resistance patterns

Among *S. aureus* isolates, the prevalence of Methicillin resistant *S. aureus* (MRSA) strains was found to be 49% (95% CI: 31, 68%). The combination of amoxicillin with *β*-lactamase inhibitor clavulanic acid (Coamox-clav) reduced the amoxicillin resistance by 42% (*P* = 27%; 95% CI: 16, 38%). Besides, the pooled estimates of amoxicillin and ampicillin resistance among CoNS isolates were 62% (95% CI: 34, 90%) and 72% (95% CI: 57, 87%), respectively. Among all antimicrobial agents tested, *S. aureus* exhibited relatively lower estimates of resistance against ciprofloxacin, 12% (95% CI: 8, 16%) and gentamicin, 13% (95% CI: 8, 18%). Likewise, ciprofloxacin had the least resistant isolates (*P* = 13%; 95% CI: 4, 23%) followed by gentamicin (*P* = 33%; 95% CI: 17, 50) for CoNS. The 3rd generation cephalosporin ceftriaxone resistance was observed among gram-positive isolates with estimates being 36% (95% CI: 17, 55%) and 37% (95% CI: 19, 54) for *S. aureus* and CoNS, respectively (Table 4).

**Table 4:** Pooled estimates of antimicrobial resistance among Gram-positive bacteria obtained from wound samples in Ethiopia

Antimicrobial agents	Pooled estimates of resistant isolates (Proportion)			
	*S. aureus*	*CoNS*		
	Pooled ES (95% CI)	I^2^ (%)	Pooled ES (95% CI)	I^2^ (%)
AMO	0.69 (0.50, 0.87)	97.64	0.62 (0.34, 0.90)	93.05
AMC	0.27 (0.16, 0.38)	81.35	NA	–
AMP	0.76 (0.60, 0.92)	97.17	0.72 (0.57,0.87)	70.85
CIP	0.12 (0.08, 0.16)	72.39	0.13 (0.04, 0.23)	65.46
CRO	0.36 (0.17, 0.55)	97.23	0.37 (0.19, 0.54)	81.58
SXT	0.35 (0.20, 0.49)	97.64	0.49 (0.31, 0.66)	75.21
ERY	0.34 (0.22, 0.46)	94.69	0.40 (0.20, 0.40)	90.05
GEN	0.13 (0.08, 0.18)	82.90	0.33 (0.17, 0.50)	88.16
MET	0.49 (0.31, 0.68)	94.50	NA	–

*AMO* Amoxicillin, *AMP* Ampicillin, *AMC* Amoxicillin-clavulanic acid, *SXT* Cotrimoxazole, *CRO* Ceftriaxone, *CIP* Ciprofloxacin, *GEN* Gentamicin, *MET* Methicillin, *ERY* Erythromycin, *NA* Not analyzed: CoNS, Coagulase negative Staphylococci

Regarding the AMR profiles of gram-negative pathogens, *E. coli* isolates exhibited the highest point estimate of resistance against ampicillin (*P* = 84%; 95% CI: 76, 91%) followed by amoxicillin (*P* = 73%; 95% CI: 63, 83%). Gentamicin and ciprofloxacin showed relatively lower estimates of resistance with pooled prevalence being 24% (95% CI: 16, 33%) and 27% (95% CI: 16, 37%), respectively. Amoxicillin-clavulanic acid combination reduced the amoxicillin resistance of *E. coli* by 16% (*P* = 57%; 95% CI: 44, 70%). Nearly half (*P* = 45%; 31, 60%) and more than half (*P* = 53%; 95% CI: 43, 64%) of *E. coli* isolates were found resistant to ceftriaxone and cotrimoxazole, respectively in wound samples in Ethiopia (Table 5).

**Table 5:** Pooled estimates of antimicrobial resistance among Gram-negative bacterial isolates obtained from wound samples in Ethiopia

Antimicrobials	Pooled estimates of resistant isolates (Proportion)							
	*E. coli*		*P. aeruginosa*		*K. pneumoniae*		*P. mirabilis*	
	ES (95% CI)	I^2^ (%)	ES (95% CI)	I^2^ (%)	ES (95% CI)	I^2^ (%)	ES (95% CI)	I^2^ (%)
AMO	0.73 (0.63, 0.83)	55.70	0.87 (0.82, 0.92)	0.00	0.90 (0.83, 0.97)	43.97	0.75 (0.57, 0.93)	86.81
*AMC*	0.57 (0.44, 0.70)	74.51	0.77 (0.63, 0.91)	63.67	0.67 (0.51, 0.83)	78.01	0.45 (0.36, 0.54)	19.01
AMP	0.84 (0.76, 0.91)	75.08	0.95 (0.92, 0.99)	0.00	0.88 (0.83, 0.93)	24.27	0.78 (0.70, 0.85)	39.67
CIP	0.27 (0.16, 0.37)	86.99	0.16 (0.09, 0.24)	87.63	0.29 (0.15, 0.42)	85.50	0.12 (0.06, 0.19)	73.68
CRO	0.45 (0.31, 0,60)	89.81	0.58 (0.35, 0.82)	95.86	0.57 (0.39, 0.75)	92.32	0.43 (0.24, 0.63)	94.04
SXT	0.53 (0.43, 0.64)	75.91	0.76 (0.68, 0.85)	51.82	0.64 (0.51, 0.77)	76.58	0.56 (0.39, 0.72)	88.19
GEN	0.24 (0.16, 0.33)	93.09	0.18 (0.11, 0.26)	66.47	0.37 (0.22, 0.52)	89.66	0.21 (0.12, 0.30)	74.31

*AMO* Amoxicillin, *AMP* Ampicillin, *AMC* Amoxicillin-clavulanic acid, *SXT* Cotrimoxazole, *CRO* Ceftriaxone, *CIP* Ciprofloxacin, *GEN* Gentamicin

Though it has become clinical concerning, the difficult to treat gram-negative bacteria *P. aeruginosa* showed the lowest pooled estimates of resistance against ciprofloxacin (*P* = 16%; 95% CI: 9, 24%, I^2^ = 87.63). Only 10% reduction in overall resistance was observed in amoxicillin-clavulanate (*P* = 77; 95% CI: 63, 91%) compared to amoxicillin alone (*P* = 87%; 95% CI: 82, 92%) in *P. aeruginosa* isolates.. More than half of *P. aeruginosa* isolates (*P* = 58%; 95% CI: 35, 82%) developed resistance to ceftriaxone. Moreover, around three-fourths of these isolates (*P* = 76%; 95% CI: 68, 85%) were found resistant to cotrimoxazole (Table 5).

The AMR profiles of the two more gram-negative aerobic bacilli (*K. pneumoniae* and *P. mirabilis*) were also tested against seven antimicrobial agents. The antimicrobials with highest estimates of resistance were found to be amoxicillin and ampicillin in both bacterial isolates. Ciprofloxacin followed by gentamicin were antimicrobials having lower point estimates of resistance in both types of isolates. More than half of *K. pneumoniae* isolates exhibited ceftriaxone resistance (*P* = 57%; 95% CI: 39, 75%) (Table 5).

#### Publication bias

**Fig. 10:**
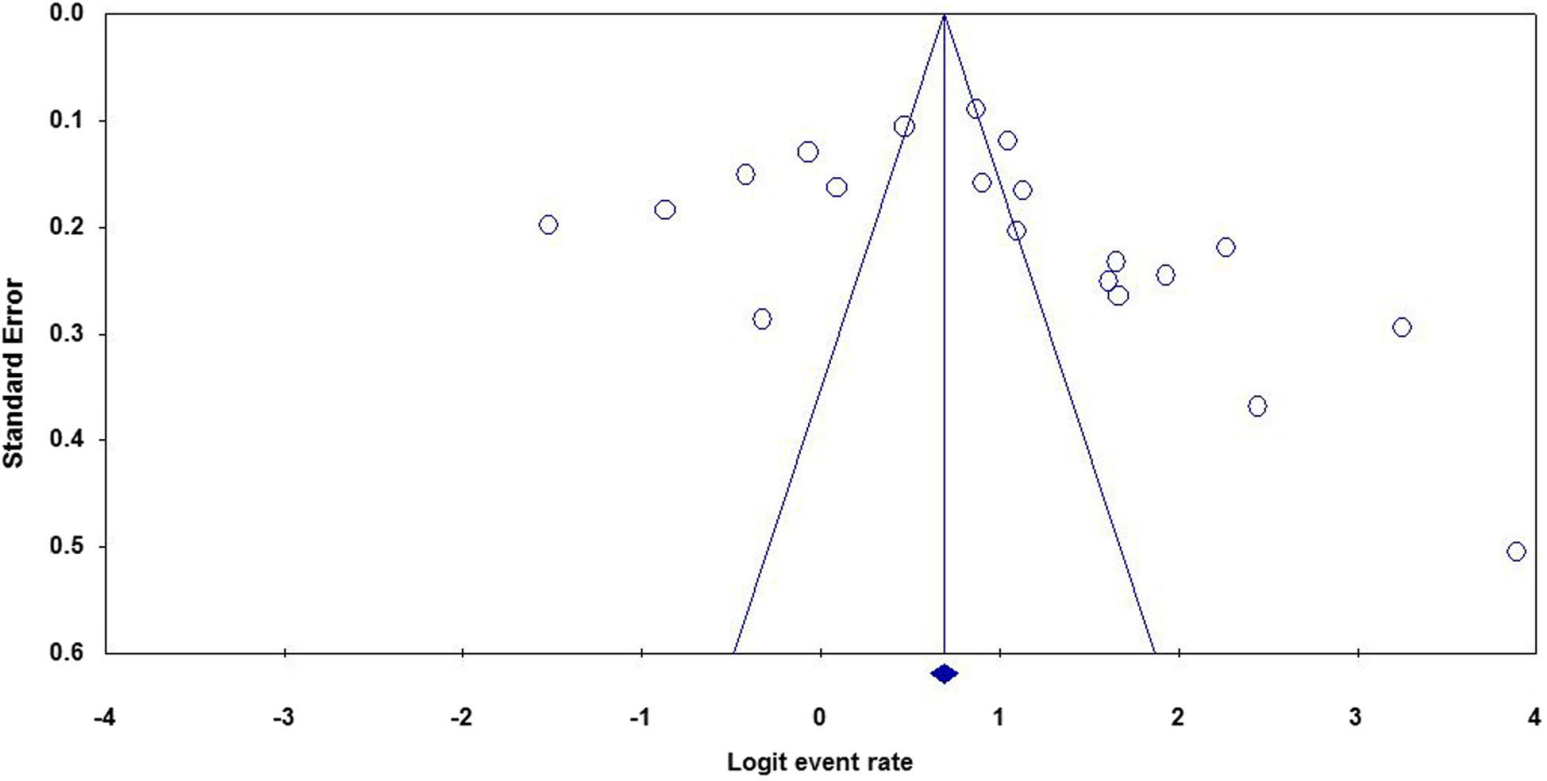
Funnel plot showing publication bias of included studies

Funnel plots of standard error with log of the prevalence of positive bacterial cultures revealed that there is some evidence of publication bias (Begg’s and Mazumdar’s test, *p*-value =0.0067; Egger’s test, *p*-value = 0.048) (Fig. 10).

## Discussion

In this systematic review and meta-analysis, more than two-thirds of wound samples (70%) were found culture positive. The subgroup analysis has also indicated higher culture positivity of burn and other non-surgical wounds compared to surgical wounds regardless of prophylactic antibiotic status. In concordant with this, a five year study conducted by Agnihotri et al reported high culture positivity (96%) from burn wound infections [6]. Besides, a summary of wound infection in orthopedics revealed that the overall culture positivity rate was estimated to be 53% [45]. The lower culture positivity of surgical wounds, as observed in subgroup analysis, might be due to the nature of surgical care (i.e. irrigation, debridement and use of prophylactic antibiotics in some cases). The most common bacterial isolate recovered from wound infection was *S. aureus* with pooled estimate being 36% which is by far higher than the prevalence of CoNS, *E. coli*, *P. aeruginosa*, *K. pneumoniae* and other gram-negative aerobic bacilli. This is further supported by several published studies which reported that gram-positive aerobic cocci, particularly, *Staphylococci*, have been the primary cause of wound infections [3–5, 7–9, 46–50]. More specifically, *S. aureus* has been a single most common pathogen isolated from several wound samples [46–49, 51]. With regard to gram-negative pathogens, the highly prevailing clinically relevant bacterial isolates were aerobic bacilli under Enterobacteriaceae including *E. coli*, *P. aeruginosa*, *K. pneumoniae*, and *P. mirabilis*. This finding is in trajectory with review of recent literature in different settings [14, 45, 48, 52].

Though it seems controversial, many studies indicated that the two highly prevalent bacterial isolates recovered from chronic wound infections were *S. aureus* and *P. aeruginosa* [3, 12, 15, 49, 51, 53, 54]. Nevertheless, a review report conducted by Macedo and Santos on burn wound infections showed that *S. aureus* was the most prevalent in the first week of wound infection with an overall prevalence of about 28.4%. It was, however, surpassed by *P. aeruginosa* from the third week onwards [55]. On the top of these, *S. aureus* and *P. aeruginosa* are opportunistic pathogenic bacteria and are widely known to cause chronic biofilm-based wound infections [51]. These pathogens are capable of making a strong biofilm that maintains the chronic infection which impairs wound healing [3, 53]. To this end, a meta-analysis of published data revealed that about 78% of non-healing chronic wounds harbor biofilms, with prevalence rates varying between 60 and 100% [12].

The overall prevalence of MRSA strains was found to be 49% indicating one in every two *S. aureus* isolates were characterized as MRSA strains. This is a clear direction of how high the healthcare settings become a reservoir of resistant strains like MRSA. Likewise, a summary of wound infections in orthopedics indicated that *S. aureus* constituted about 34% of all isolates and 48% of this isolate were reported to be MRSA [45]. Conditions such as hospitalization, prophylactic use of antimicrobials in surgical procedures and use of broad spectrum antimicrobials predispose patients for such resistant strains [3]. The rapid development and spread of MRSA clones across the globe often results in such hospital outbreaks [56, 57].

Due to β- lactamase producing capability and modification of penicillin binding proteins*, S. aureus* has developed resistance to majority of β-lactam antibiotics (mainly penicillins and cephalosporins). As the analysis tried to address, the third generation cephalosporin ceftriaxone resistance was estimated to be 36%. Though it is less pathogenic than *S. aureus*, CoNS had also developed closer estimates of resistance in these antibiotics. Most notably, the steady erosion of the effectiveness of β-lactam antibiotics for treatment of *S. aureus* infection is due to the fact that it’s extraordinary adaptability in developing resistance with several mechanisms [10, 56, 57]. Gram-positive cocci particularly *Staphylococci* such as *S. aureus* produce β-lactamase enzymes which in turn degrade the β-lactam ring of majority of β-lactam antibiotics (penicillins, cephalosporins, carbapenems and monobactams) and make these drugs devoid of any antibacterial activity. Though they have a wide range of bacterial coverage, the β-lactamase sensitivity of broad (ampicillin and amoxicillin) and extended spectrum (antipseudomonal) penicillins are no longer effective for β-lactamase producing *Staphylococci* such as *S. aureus* [6, 14, 56, 58]. To this end, the use of combination of β-lactamase enzyme inhibitors (e.g. clavulanic acid, sulbactam, tazobactam) with these sensitive β-lactam antibiotics has been somehow a notable strategy in combating resistance against strains constitutionally producing these enzymes. In this study, combination of amoxicillin with clavulanic acid (Coamox-clav) reduced the pooled estimates of amoxicillin resistance from 69 to 27% clearly indicating the involvement of these enzymes in resistance development. This finding is in trajectory with a 5-year study conducted by Agnihotri et al. in which *S. aureus* showed high estimate of resistance (about 75%) to standard β-lactam antibiotics whereas coamox-clav resistance was reduced to 23.5% [6].

Moreover, *S. aureus* has also developed resistant to β-lactamase resistant penicillins (methicillin, naficillin, oxacillin, cloxacillin and diclocoxacillin) with apparently different mechanism. From experience, resistant to these agents was historically treated as MRSA since the first agent in this class of penicillins is methicillin [59–61]. *S. aureus* acquires resistance genes which encode a modified form of penicillin binding proteins (PBP). In MRSA, the horizontally acquired *mecA* gene encodes PBP2A. PBP2 is the only bifunctional PBP in *S. aureus*. In MRSA strains, the essential function of PBP2 may be replaced by PBP2A which functions as a surrogate transpeptidase. Since almost all β-lactams have little or no affinity to this protein, cross resistance occurs regardless of β-lactamase stability status [57, 60]. This is a likely justification why more-than one-thirds of *S. aureus* strains were found resistant to ceftriaxone. Apart from this, macrolides (erythromycin) and sulfa drugs (cotrimoxazole) resistance was observed in about one-thirds of *S. aureus* strains. Generally, this study showed that more than 10% of *S. aureus* isolates were also resistance to ciprofloxacin and gentamicin. CoNS had also showed higher gentamicin resistance than *S. aureus*.

Observing the AMR profile of gram-negative bacteria, a high estimate of resistance was observed in β-lactam antibiotics. All gram-negative bacilli have developed clinically significant resistant against broad spectrum penicillins (amoxicillin and ampicillin). The combination of β-lactamase inhibitor clavulanic acid with amoxicillin did not show an appreciable reduction. The Coamox-clav resistance in *P. aeruginosa* was about 77%. Likewise, more than 50% of *P. aeruginosa* and *K. pneumoniae* isolates had developed resistance to the third generation cephalosporin ceftriaxone. From sulfa drug combination, cotrimoxazole resistance was observed in more than half of all isolates in each gram-negative bacterium with the highest resistance estimate (76%) observed in *P. aeruginosa*. Even if the degree of susceptibility varies across strains, relatively lower estimates of resistance were observed in ciprofloxacin and gentamicin making these agents relatively effective for treatment of wound infections.

In this regard, a plasmid encoded extended-spectrum β-lactamases (ESBLs) production has been alarmingly increasing in gram-negative bacteria particularly in *Enterobacteriaceae* [62–64]. A study indicated that among *K. pneumoniae* and *E. coli* isolated from wound samples, around 11% produced ESBL (12.2 and 10.3% for *K. pneumoniae* and *E. coli*, respectively) [62]. ESBLs have the ability to hydrolyze extended spectrum β-lactams including third generation cephalosporins (e.g. ceftriaxone, ceftazidime and cefotaxime) and the monobactams (aztreonam). Carbapenems have been the treatment of choice for serious infections due to ESBL-producing organisms, yet carbapenem-resistant isolates have recently been reported [63, 65–67].

The difficult to treat gram-negative bacteria *P. aeruginosa* has naturally known to develop resistance by its outer membrane (porine channels) permeability problems coupled to adaptive mechanisms such as efflux pumps and can readily achieve clinical resistance. Almost all penicillins, except the fourth generation series (antipseudomonal agents such as ticarcillin and piperacillin), are not clinically effective at all for the treatment of *Pseudomonas* infections.. Likewise, majority of the third generation cephalosporins and all of the prior generations are no longer effective since they are not able to penetrate the porine channels of this bacterium though other resistance mechanisms should also be considered. Generally, this bacterium is highly notorious to develop resistance to multiple antibiotics and has joined the ranks of ‘superbugs’ due to its enormous capacity to engender resistance [48, 68–73].

Historically, the classes of antibiotics used in the treatment of wound infections include the β -lactams and aminoglycosides [47]. At present, guidelines have indicated that antibiotics prescribing practice primarily relies on expert opinion not on evidence based medicine. This has now created difficulties in interpreting and implementing it in clinical settings [15]. Besides, inappropriate use of antibiotics for surgical prophylaxis increases both the cost and the selective pressure favoring the emergence of resistant strains. Published reports indicated that, from about 30–50% of antibiotics being used for surgical prophylaxis, between 30 and 90% of it is potentially inappropriate [16, 74, 75]. The meta-analysis has vividly indicated that wound is contaminated with multiple microorganisms (poly-microbial infection) and hence antibiotic treatment should be tailored according to the microbial profiles. Particularly, *S. aureus* and *P. aeruginosa* are among the pathogen of interest and have several resistance mechanisms making the treatment challenging. In general, the most common drugs used to treat *S. aureus* infections include amoxicillin/clavulanate, ampicillin/sulbactam, and cloxacillin whereas MRSA strains are better treated with levofloxacin, vancomycin, linezolid and tigecycline. In the presence of co-infections related to *S. aureus* and *P. aeruginosa*, piperacillin/tazobactam, carbapenems and ciprofloxacin may represent the first choice for treatment of wound infections [53].

## Conclusion

Generally, the wound culture positivity was very high indicating the likelihood of poly-microbial load. Surgical wounds had relatively lower culture positivity than non-surgical counterparts though the role of prior local wound care following surgery is yet to be investigated. *Staphylococci* have been the predominant gram-positive cocci in wound infection with *S. aureus* being by far the most prevalent isolate. This finding alarms the high load of MRSA strains in hospital settings as well. Gram-negative aerobic bacilli under *Enterobacteriaceae* are also commonly isolated pathogens in wound infections. Though it is expected in *S. aureus* and *P. aeruginosa*, all isolates exhibited highest estimates of resistance against β-lactam antibiotics. Most notably, resistance to the 3rd generation cephalosporin ceftriaxone was observed in more than 40% of all gram-negative isolates (more than half in *K. pneumoniae* and *P. aeruginosa*). Though more than 10% of resistance was reported in all isolates, ciprofloxacin and gentamicin were relatively good in treating wound infections with poly-microbial etiology. Hence, considering microbial epidemiology and AMR patterns, treatment should be tailored for addressing poly-microbial etiology thereby effectively manage wound infection and its complication as well as preserve antimicrobials and contain AMR.

### Limitation of the study

Even if the study has extensively included all relevant data regarding wound infection, it was not without limitations. There was no clear demarcation of wound samples in inpatient and outpatient settings. Besides, there was no documented history of prophylactic use of antibiotics in individual studies though we conducted subgroup analyses to reduce culture positivity/negativity errors in surgical patients. The antimicrobial susceptibility testing was a little bit different across studies. Therefore, this meta-analysis should be seen in the context of such limitations.

## Additional files

Additional file 1: **Table S1**. Completed PRISMA checklist: The checklist highlights the important components addressed while conducting systematic review and meta-analysis from observational studies (DOC 66 kb)

Additional file 2: **Table S2.** Data abstraction format with crude data: The table presented the ways of data collection (study characteristics and outcome measures) in Microsoft excel format. (XLSX 38 kb)

## Data Availability

All the data is contained within the manuscript and additional files.

## Abbreviations

AMR: Antimicrobial Resistance
CoNS: Coagulase Negative Staphylococci
ESBLs: Extended Spectrum β-lactamases
JBI: Joanna Briggs Institute
MeSH: Medical Subject Headings
MRSA: Methicillin Resistant *Staphylococcus aureus*
PBP: Penicillin Binding Protein
PRISMA: Preferred Reporting Items for Systematic review and Meta-Analysis
